# A PRISMA-compliant systematic review of the endpoints employed to evaluate symptomatic treatments for primary headaches

**DOI:** 10.1186/s10194-018-0920-9

**Published:** 2018-09-21

**Authors:** D. García-Azorin, N. Yamani, L. M. Messina, I. Peeters, M. Ferrili, D. Ovchinnikov, M. L. Speranza, V. Marini, A. Negro, S. Benemei, M. Barloese, on behalf of the European Headache Federation School of Advanced Studies (EHF-SAS)

**Affiliations:** 1 Headache Unit Neurology Department, Hospital Clínico Universitario Valladolid, Avda. Ramón y Cajal 3, 47005 Valladolid, Spain; 2 Danish Headache Centre and Department of Neurology, University of Copenhagen, Rigshospitalet Glostrup, Copenhagen, Denmark; 3 Headache Department, Iranian Center of Neurological Research Neuroscience Institute, Tehran University of Medical Sciences, Tehran, Iran; 4 Child Neuropsychiatry School, University of Palermo, Palermo, Italy; 5 U.O. Neuropsychiatry - ARNAS Civico, PO Di Cristina, Palermo, Italy; 6 Neurology Department, University Hospital of Brussels, Brussels, Belgium; 7 Headache Center, Bambino Gesù Children Hospital IRCCS, Rome, Italy; 8 Pavlov First Saint Petersburg State Medical University, Saint Petesburg, Russia; 9 Almazov National Medical Research Centre, Saint Petesburg, Russia; 10 Internal Medicine Department, Sant’Andrea Hospital, Rome, Italy; 11 Regional Referral Headache Centre, Sant’Andrea Hospital, Department of Clinical and Molecular Medicine, Sapienza University, Rome, Italy; 12 Headache Centre, Careggi University Hospital, University of Florence, Florence, Italy; 13 Danish Headache Center, Department of Neurology, Rigshospitalet-Glostrup, University of Copenhagen, Copenhagen, Denmark; 14 Department of Clinical Physiology and Nuclear Medicine, Center for Functional and Diagnostic Imaging, Hvidovre Hospital, Copenhagen, Denmark

**Keywords:** Primary headaches, Clinical trials, Acute, Triptans, Non-steroidal anti-inflammatory, Prisma-guidelines, Endpoints

## Abstract

**Background:**

Primary headache are prevalent and debilitating disorders. Acute pain cessation is one of the key points in their treatment. Many drugs have been studied but the design of the trials is not usually homogeneous. Efficacy of the trial is determined depending on the selected primary endpoint and usually other different outcomes are measured. We aim to critically appraise which were the employed outcomes through a systematic review.

**Methods:**

We conducted a systematic review of literature focusing on studies on primary headache evaluating acute relief of pain, following the PRISMA guideline. The study population included patients participating in a controlled study about symptomatic treatment. The comparator could be placebo or the standard of care. The collected information was the primary outcome of the study and all secondary outcomes. We evaluated the studied drug, the year of publication and the type of journal. We performed a search and we screened all the potential papers and reviewed them considering inclusion/exclusion criteria.

**Results:**

The search showed 4288 clinical trials that were screened and 794 full articles were assessed for eligibility for a final inclusion of 495 papers. The studies were published in headache specific journals (58%), general journals (21.6%) and neuroscience journals (20.4%).

Migraine was the most studied headache, in 87.8% studies, followed by tension type headache in 4.7%. Regarding the most evaluated drug, triptans represented 68.6% of all studies, followed by non-steroidal anti-inflammatories (25.1%). Only 4.6% of the papers evaluated ergots and 1.6% analyzed opioids.

The most frequent primary endpoint was the relief of the headache at a determinate moment, in 54.1%. Primary endpoint was evaluated at 2-h in 69.9% of the studies. Concerning other endpoints, tolerance was the most frequently addressed (83%), followed by headache relief (71.1%), improvement of other symptoms (62.5%) and presence of relapse (54%). The number of secondary endpoints increased from 4.2 (SD = 2.0) before 1991 to 6.39 after 2013 (*p = 0.001*).

**Conclusion:**

Headache relief has been the most employed primary endpoint but headache disappearance starts to be firmly considered. The number of secondary endpoints increases over time and other outcomes such as disability, quality of life and patients’ preference are receiving attention.

## Background

Primary headaches are the most prevalent neurological disorders and the main neurological cause of years lived with disability, particularly in the middle age group under 50 years old adults, in which migraine is the first cause of disability [1]. They represent also one of the main neurological disorders regarding economical costs [2], and symptomatic treatment accounts for the biggest part of them [3].

Also for trials, success is a matter of perspective and depends mainly on expectations. An intervention is considered efficacious if it reaches a predefined endpoint, thus the careful definition of every endpoint is critical. Not only concerning patients’ satisfaction and relief, but also in order to reach the approval from the regulatory authorities, such as the European Medicines Agency (EMA) and the Food and Drug Administration (FDA).

In the field of headache, traditional endpoints in chronic headaches have been related to the decrease in number of days with headache, changes related to pain intensity, analgesics uptake and emergency department visits. Nowadays novel factors such as quality of life or work absenteeism begin to be taken into account.

Regarding symptomatic therapies, many outcomes have been proposed. The International Headache Society (IHS) created guidelines in order to harmonize studies evaluating acute treatment. The first version was published in 1991 [4], a second edition arrived in 2000 [5] and the last dates from 2012 [6]. Every edition added new proposed outcomes and instructions about how every endpoint should be measured. Table 1 presents the list of outcomes mentioned in the three versions of IHS Guidelines.

**Table 1:** Recommended endpoints from IHS guidelines for migraine drug trials according to the edition

1st edition (published in 1991)	2nd edition (published in 2000)	3rd edition (published in 2012)
**Number of attacks resolved within 2h**	**Pain-free after 2h**	**Percentage of patients free of pain at 2h**
		Incidence of relapse
Duration of headache	Sustained pain-free 24h	Sustained pain freedom
		Total migraine freedom
Severity of headache	Headache intensity	Intensity of headache
Global rating of attack severity	Disability	Headache relief
		Time to meaningful relief
		Time to pain freedom
Escape medication	Rescue medication	Rescue medication
Global evaluation of medication	Global evaluation of medication	Global evaluation of medication
		Global impact (disability and quality of life)
Presence of nausea and vomiting		Migraine-associated symptoms
	Adverse events	Adverse events
	Patients preference	Preference to treatment
		Treatment of relapse
	Consistency of effect.	

In bold, the recommended primary endpoint

As shown in Table 1, the recommended main endpoint has changed over time from headache relief to complete pain freedom. This was motivated by the fact that placebo response in headache relief could be substantial, over 30%, whereas pain freedom placebo response is just around 9% [7]. Despite their utility, adherence to these guidelines seemed to be low, just 31% of the studies addressing acute treatment ascribed to them in the 2002–2008 period [8].

Patient preference has received little and intermittent attention. The triptan-era brought many studies trying to assess what patients value most, and complete relief of pain, a fast onset of action and lack of recurrence were the preferred endpoints. Non-clinical endpoints such as productivity, disability, direct costs, and quality of life have also been considered recently [9], considering headache as a multidimensional disease.

As we mentioned above, the criteria for success have been defined by expert consensus, and patients’ opinions are not usually considered. The concept of Patient Related Outcome Measure (PROM) defines self-reported measures about symptoms, functional status and perceptions from a patient perspective [10]. This novel approach is receiving growing attention and identifying patients’ preferences might help to create realistic outcomes, considering patients expectations [11, 12].

We aimed to critically appraise the employed outcomes by conducting a systematic review. The study population included patients with acute primary headache; we considered all the possible interventions pursuing the resolution of the headache episode. Studies employed placebo or the standard of care as comparator. We analyzed every employed outcome.

## Methods

We conducted a systematic review of literature focusing on studies on primary headache addressing the acute relief of pain, following the PRISMA guidelines [13].

### Search criteria

The target population was primary headache sufferers, the study population included patients participating in a controlled study about the symptomatic treatment for the relief of the headache episode. The intervention was compared with placebo or the standard of care. The collected information was the employed outcome of the study, differentiating between primary and secondary endpoints.

We did not restrict the search in time, considering all available articles until the search. We reviewed studies in all the European languages. Age of participants or the country in which the study took place was not limited.

We included studies addressing the acute treatment for primary headaches providing information about primary and secondary endpoints. The inclusion criteria were: 1) clinical trials comparing with placebo or standard of care, 2) studies conducted in humans, 3) with full text availability.

We excluded studies if they were: 1) not original researches, 2) not focusing on treatment and addressing other issues, 3) studying therapies that are not suitable for self administration by patients, such as intravenous therapies, 4) performed on emergency department setting, 5) not providing information about the relief of pain, 6) focused on secondary headaches.

### Search strategy

The employed source was MEDLINE database. The search was performed on April 22nd 2018 and included all the studies. The electronic strategy of search was designed in order to include the primary headaches and all the possible acute therapies.

The strategy of search combined terms such as primary headache, migraine, tension-type, trigeminal, hemicrania, cluster, Short-lasting Unilateral neuralgiform headache with conjunctival injection and tearing (SUNCT) and Short-lasting unilateral neuralgiform headache attacks with cranial autonomic symptoms (SUNA) by the use of boolean operators, combining with the available therapies. We employed truncations and wildcards to optimize the search. The employed command is available in Appendix section. We included common analgesics such as acetaminophen/paracetamol or metamizol, non-steroidal anti-inflammatory drugs (NSAIDs), triptans, anti-emetics (if employed for pain relief), opioids, ergot derivatives, caffeine, magnesium, oxygen, devices that could be easily employed by patients at home and novel drugs such as lasmiditan or gepants, screening all the possible results.

#### Database creation and included variables

We created a database with all the potential studies. Two investigators peer reviewed independently all the abstracts and selected them according to inclusion and exclusion criteria. Results were compared and in cases with lack of agreement the rest of the team decided by consensus if the study should be included or not. After that, a different investigator reviewed the full document, addressing the final eligibility if information about the endpoint was provided and excluding the paper in the opposite case.

As we were not specifically reviewing the results of the interventions, we listed the different interventions considering the different primary and secondary outcomes employed in each study, the year of publication and the evaluated drug. We generated an electronic database employing Microsoft Excel.

We considered the risk of publication bias so we included only the information contained in the material and methods section or in the study protocol, in order to avoid lack of information. We tried to minimize the bias of missing articles using a wide search strategy, employing MeSH terms in the search and fully evaluating many of the papers in order to obtain firm evidence.

### Statistical description and analysis

We present the data as frequency for categorical variables and means and their standard deviation or medians and Inter Quartile Range (IQR) for quantitative data. For analytical purposes, we classified the journals into three groups: Headache specific journals, neuroscience journals and general medicine journals. All the journals included in each group can be consulted in the Appendix. We also divided the time into 4 periods considering the date when the IHS official guidelines for symptomatic studies were published, being the intervals: before 1991, 1992–2000, 2001–2012 and after 2013. We employed SPSS v20.0 IBM for the statistical analysis employing the pertinent test for each type and distribution of variable.

## Results

**Fig. 1:**
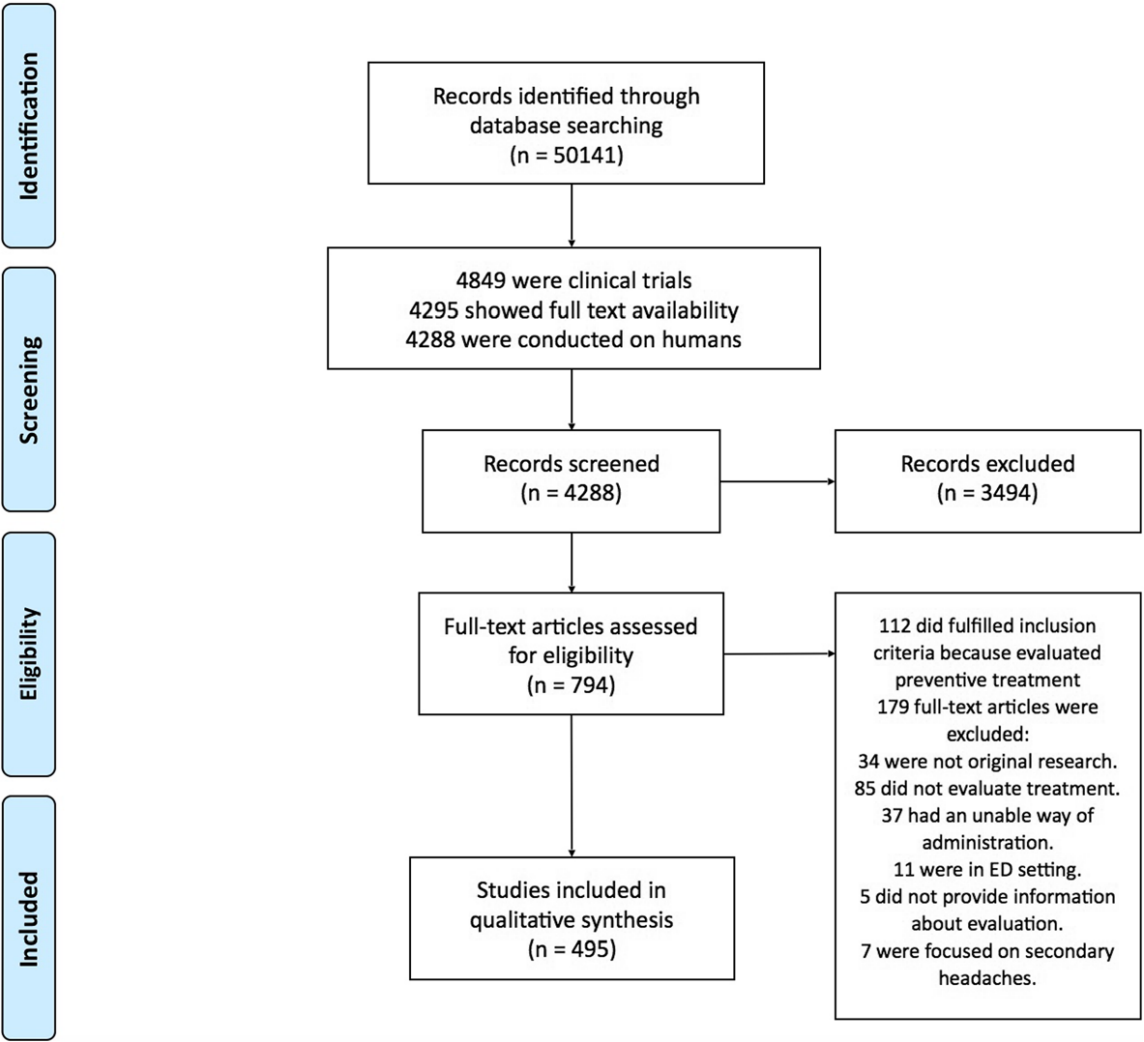
PRISMA 2009 guidelines flow chart showing the flow of the search and analysis

We present the number of identified articles, those screened and those that fulfilled inclusion and exclusion criteria in the PRISMA flow chart (Fig. 1).

We included 495 papers in the final analysis. 58% of the studies were published in headache specific journals, 20.4% in neuroscience journals and 21.6% in general journals. The specific journals that showed most articles were *Headache* with 156 papers, *Cephalalgia* with 112, *Neurology* 36, *European Neurology* 19 and *Journal of Headache and Pain* with 15.

**Fig. 2:**
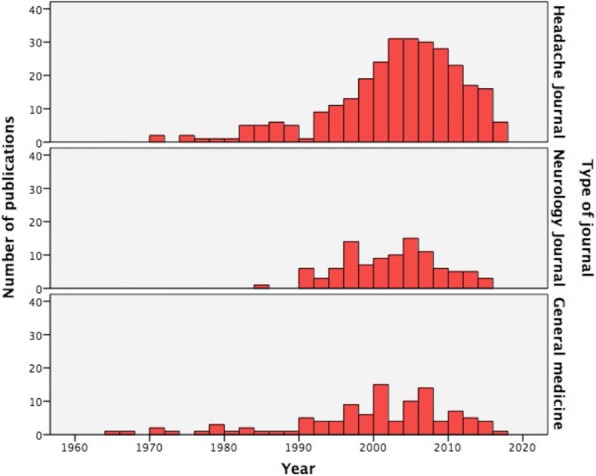
Number of publications per year depending on the type of journal. On the upper part, Headache specific journals, in the middle Neurology journals and in the lower part General medicine journals. Y-axis represent the number of publications and X-axis the year

Regarding the year of publication, the mode was 2005 and the median was 2003, with decreasing the number of papers after 2010 (Fig. 2). The pattern of publication differed comparing Headache Journals, which showed a median year of publication of 2004 (IQR 1998–2009) and general neurology journals, which had a median year of 2003 (IQR 1997–2006) and general medicine journals, whose distribution had a median of 2001 (IQR 1995–2007), *p = 0.05*. (Fig. 2).

### Addressed headaches

The evaluated headache according IHS classification were migraine in 87.8%, with 22 studies addressing specifically menstrual migraine, 4.7% of the studies were focused on tension type headache, 3.6% analyzed trigeminoautonomal cephalalgias, 2 studies included patients with both migraine and tension type and 19 studies mentioned primary headaches without additional specifications. We did not find any trial on patients with other primary headaches.

### Studied drugs

The most frequently evaluated group of drugs was triptans, representing 68.6% of the studies. Sumatriptan was the most studied, counting 115 papers and representing 45,1% of the triptan articles, followed by rizatriptan (37 studies, 14.5%), zolmitriptan (33, 12.9%), almotriptan (27, 10.8%), eletriptan (20, 7.8%), frovatriptan (10, 3.9%), naratriptan (6, 2.4%), avitriptan (3, 1.2%) and 4 studies did not specify the triptan clearly.

Non-steroidal anti-inflammatories were analyzed in 25,1% of the studies. Naproxen was the most frequently evaluated, in 31 studies (26,9% of NSAID studies), followed by acetylsalicylic acid in 27 (23.5%), ibuprofen in 17 (14.8%), diclofenac in 10 (8,7%) and dexketoprofen and COX-2 inhibitors with 9 studies each (7.8%). Paracetamol or acetaminophen were evaluated in 7.4% of the studies. Only 4.6% of the papers analyzed ergots and 1.6% studied opioids. Gepants were evaluated in 10 studies and only 1 Lasmiditan matched our criteria.

**Fig. 3:**
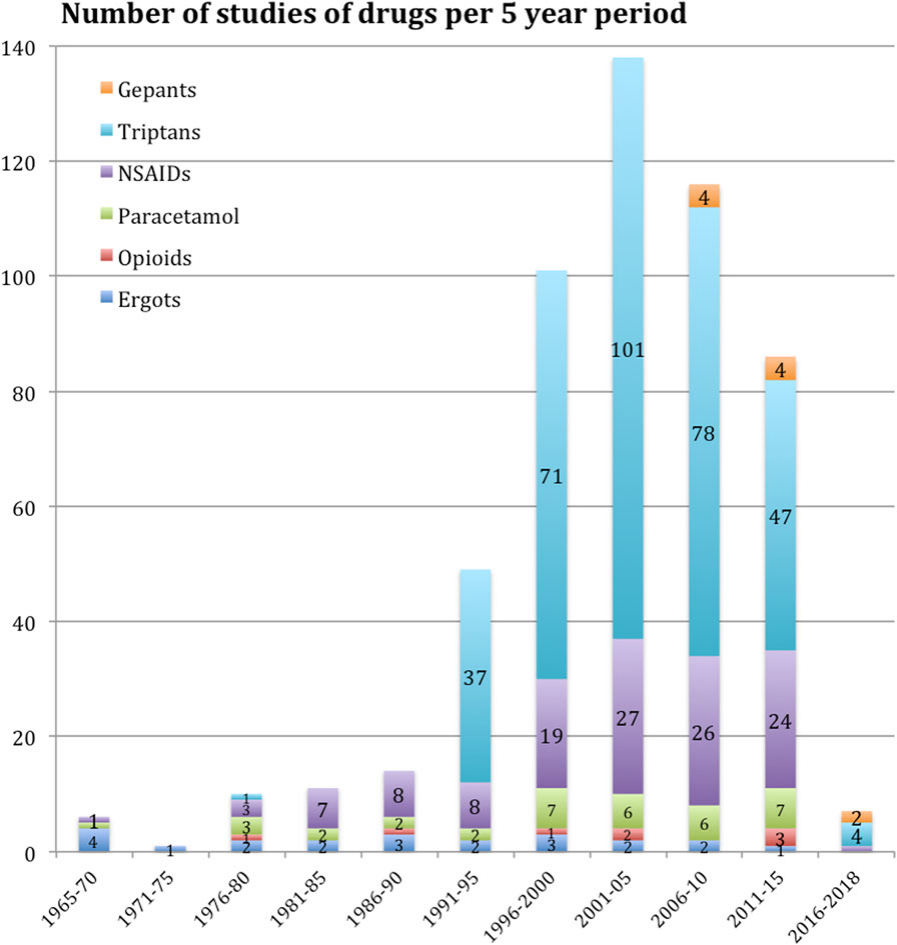
Number of studies evaluating different pharmacological groups. X-axis represents the time period and Y the number of studies

Up to 30.9% of the studies evaluated at least two different analgesics, naproxen-sumatriptan being the most frequent combination in 18 studies and dexketoprofen-frovatriptan in 4. 70.6% of the studies included a placebo arm. Figure 3 presents the number of publications per 5-year period differentiating the studied drug.

### Primary endpoints

**Fig. 4:**
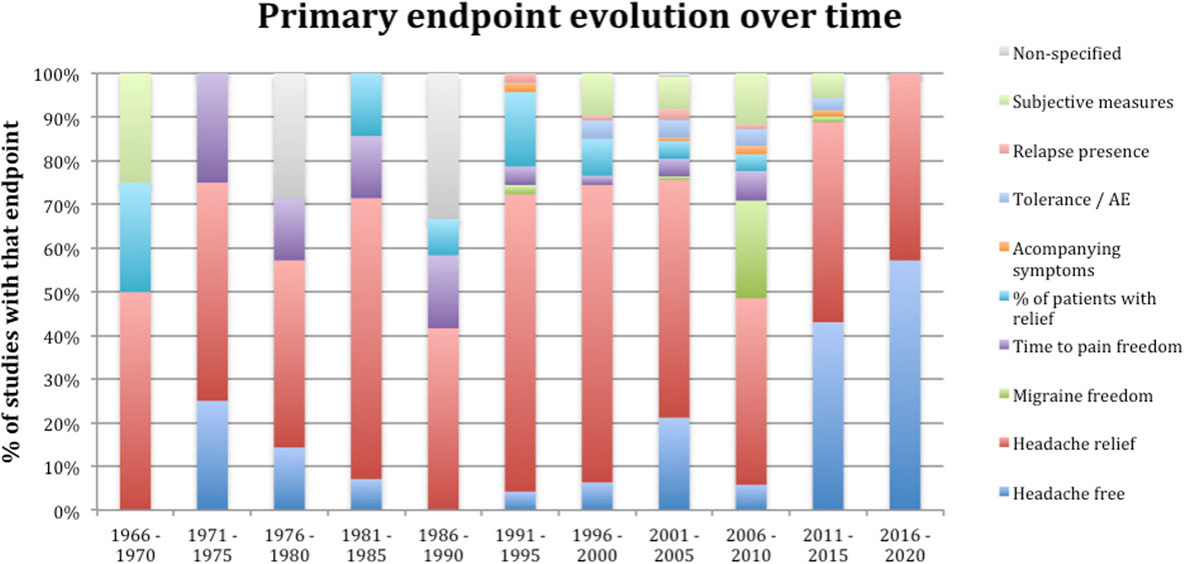
Percentage of studies with a determinate primary endpoint per 5-year periods. Y-axis shows the percentages per each endpoint of the total studies of each period, X-axis shows the different periods. AE = Adverse Events

Concerning the primary endpoints, the most frequent endpoint was the relief of the headache at a determinate moment, representing 54.1% of the studies, followed by the complete disappearance of the headache at a determinate time in 16.2% of the studies, subjective variables in 7.2%, the percentage of patients with relief of the headache in 5,9%, total migraine freedom in 5.3%, time to the pain free situation in 4.5% of the studies, tolerance and adverse event presence in 3.1%, relapse of the headache in 1.2% of the papers, 1% focused on the accompanying symptoms and 2.8% of the papers did not specified clearly the primary endpoint. Figure 4 represents the evolution of the primary endpoint over time, divided in 5-year intervals and showing the percentage of each primary endpoint in each time frame.

**Fig. 5:**
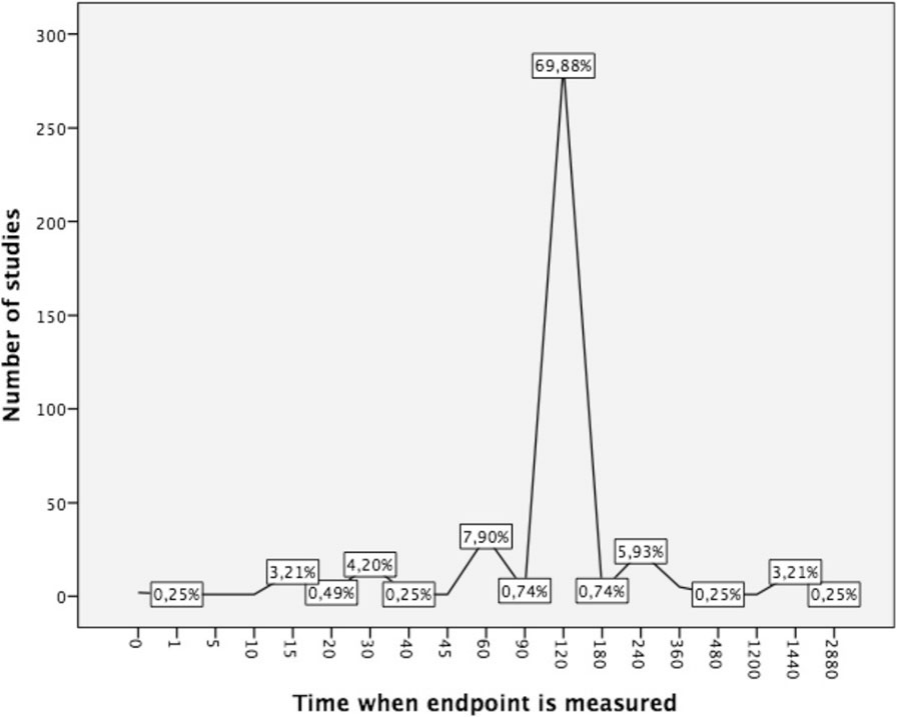
Percentage of studies evaluated at each determined time. X-axis shows the predefined time points. Y-axis represents the number of papers. Percentage of the total appears squared

The time when the primary endpoint was evaluated was 120 min in 69.9% of the studies. In studies evaluating tension type headache and migraine, the percentage ascended to 74.6%. 65.3% of the Neuroscience journals analyzed the primary endpoint at 120 min, in comparison with 58.9% of the Headache journals and 44.9% of the General Medicine journals (*p = 0.008*). Figure 5 presents the percentage of studies that employed each time point in the evaluation of the primary endpoint.

### Secondary endpoints

**Fig. 6:**
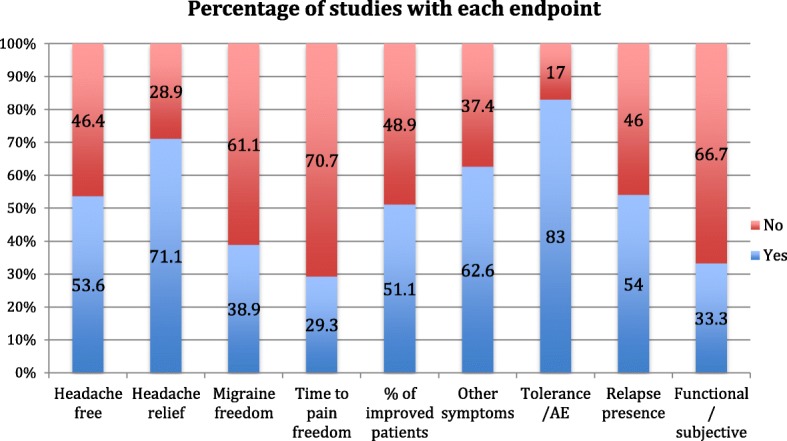
Percentage of studies that fulfilled each of the proposed endpoints. Blue represents articles that satisfied it and red the percentage of studies that did not. AE: Adverse Events

Regarding the secondary endpoints of the studies, the percentage of studies that analyzed specifically each endpoint is presented in the Fig. 6. The most frequently evaluated was tolerance and adverse event presence followed by headache relief and effect on other symptoms of the headache different to the pain.

The number of endpoints that were addressed was higher in headache specific journals, with a mean of 4.89 (SD = 2.1), followed by neuroscience journals (4.56, SD = 1.8) and general medicine journals (4.23, SD = 2.1), *p = 0.01*. That number also variated on time, showing that studies published before the first IHS recommendations included a mean of 4.20 (SD = 2.0) recommendations; after that publication and before the second edition (1991–2000) the mean number was 4.76 (SD = 1.9) and after the second IHS guidelines the mean number was 4.5 (SD = 2.0) (2000–2012 period) and after the publication of the third IHS guidelines (from 2013 to present) the mean number of fulfilled endpoints was 6.39 (SD = 2.1), (*p = 0.001*).

**Fig. 7:**
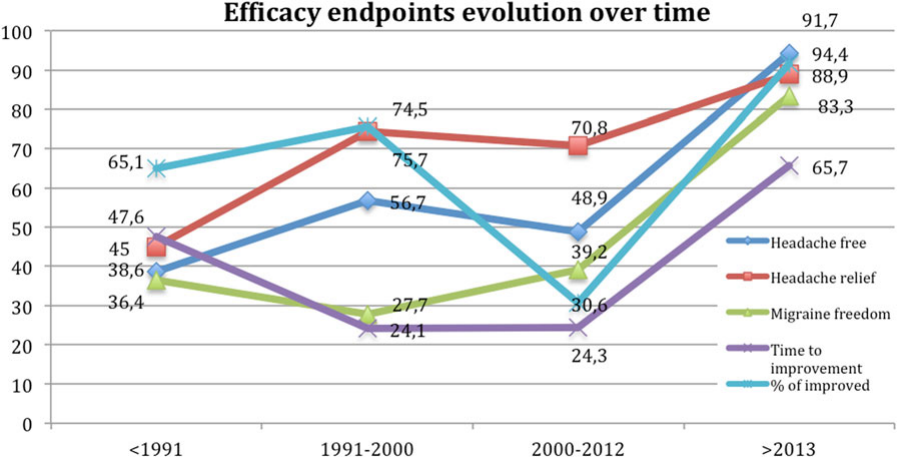
Percentage of studies addressing the recommended efficacy endpoints per period, dividing X-axis in the periods pre-IHS recommendations and after each version. Y-axis represents the percentage of studies. Considered endpoints are: complete headache resolution, headache relief, complete migraine resolution, time to the improvement, and percentage of improved patients

The percentage of studies that fulfilled all the IHS recommendations was of 10.8% for the first version, 8.2% for the second version and 4.5% for the third one. Figure 7 represents the percentage of studies that analyzed each efficacy endpoint over time. Endpoints evaluating functionality were addressed in 33% of the studies. Patients’ preference was considered only in 4.98% of the studies and the global evaluation of the drug in 5.1% of the papers.

## Discussion

In this study, we systematically reviewed all the randomized controlled studies evaluating acute therapies for the treatment of primary headaches. We addressed not only which headaches and drugs under analysis, but also the way they were evaluated, considering if IHS recommendations were fulfilled.

The vast majority of the studies took place in migraine patients. The low prevalence of other primary headaches difficult its study in controlled trials, nevertheless other prevalent conditions such as Tension Type Headache or Cluster Headache seem to be underrepresented. In line with the previous finding, migraine-specific therapies were the most frequently studied. Triptans and NSAIDs aggregated the majority of the studies, even when many other analgesics are used in other painful conditions, most of them have not been properly analyzed specifically in headache.

The most frequently used main endpoint determining the success has been headache relief in the majority of studies. We observed that recently, other endpoints proposed by the IHS guidelines start to be systematically addressed, specifically complete freedom of pain or disappearance of all migranous symptoms. Dealing with an exquisitely subjective condition as pain could be hard. The wide inter-individual variability and the high risk of placebo effect, with the subsequent possibility of false positive or false negative results, are well-known. As IHS guidelines recommend, it is desirable to employ total headache disappearance instead of relief, as the improvement implicates considerable subjectivity.

Two hours is the period defined as critical in the resolution of the headache such as migraine or tension type headache and most of the studies adhere to it. Only a minority of studies employs the time to the disappearance of the headache, probably because of the complexity in its evaluation and a more complicated statistical analysis. Many other endpoints were usually addressed, mainly tolerance, effect on other symptoms associated with the headache and relapse presence.

The number of studies clearly increases with the arrival of the triptans, multiplying the number of publications in a 10-fold in comparison with the pre-triptan era. Sumatriptan is the most studied drug so far, present in 23.2% of all the trials indexed in PubMed. In the post-triptan era we have found a progressive decrease of publication rate of studies, which could be increased in the near future with the arrival of new agents such as lasmiditan [14] and the gepants [15]. We have found also an increasing tendency over time to publish in headache specific journals in comparison with the past, when general medicine journals represented the preferred target for publication.

Concerning the cost, difficulty and bureaucracy that conducting a randomized control trial (RCT) implicates, many drugs that we use in our daily practice have not been properly studied. Nowadays, companies support most of the studies and “orphan” drugs do not attract great interest. Something similar can be noticed in headaches with a low prevalence, in which the number of existing RCT’s is low or even non-existent. In our study, almost 88% of the studies were of migraine. Funding agencies and researchers should be encouraged to evaluate drugs in orphan indications as well.

One of the most surprising findings was the low number of studies evaluating opioids, considering how frequently patients employ them in the real world studies. We only found 8 studies, representing 1,6% of all the studies. This could be partly explained because 5 additional studies were excluded because they took place in the Emergency Department Setting or they implicated administration routes such as intravenous or intramuscular. It is well known that opioid consumption has been associated with chronification of some headaches and that they are considered one of the main causes of overuse of analgesics [16, 17] nevertheless studies evaluating their efficacy in headache relief and safety are surprisingly scarce. Many of them were evaluated in combination with other drugs, the majority employed improvement of headache instead of headache relief and their adherence to IHS guidelines was even lower. As far as this study did not evaluate specifically results of the trials, we cannot defend their use, which concerning their potential risks, should be cautious until better and newer studies take place.

Despite the fact that IHS published 3 editions of the guidelines for studies evaluating symptomatic treatment, most of the studies do not follow them completely. Adherence to the guideline is important not only because it assures the quality of the study but also ensures comparability of data. For example, the time when the main endpoint was evaluated was not 120 min in up to 30% of the studies. We found a hopeful trend, as newer studies are conducted with a closer adherence to guidelines, including a higher number of endpoints and showing an increasing percentage of efficacy endpoints being measured, especially in headache specific journals, which showed statistically significant differences subscribing more IHS guidelines. It may be truth that the burden of headache is a frequent topic in headache journals; nevertheless excellence in trial designs and reports should be mandatory no matter the journal of publication.

Considering the functional limitation and quality of life impairment that most of the headaches implicate, exploring the ability to work or act normally has also been specifically evaluated. As primary headaches do not implicate mortality, the morbidity and indirect cost due to functional impairment is gaining attention, so addressing it specifically may give additional data of the positive effect of a treatment. Only a third of the studies considered this type of endpoints, so their presence still should be increased.

Personal and social burden of headache disorders is significant. Disability and health-related quality of life have been increasingly used to help patient and clinicians make better decisions regarding headache treatment and their presence in research tends to grow. Novel tools are PROMs [18] that focus on patient perspective, therapeutic preference, and satisfaction with treatments. PROMs may be an indirect indicator of efficacy, as patients will not probable feel satisfied with inefficacious or poorly tolerated treatments [19]. Nevertheless, just 10% of the published studies considered these outcomes. IHS guidelines encourage authors to employ them since the year 2000 [5] and they represent one of the strategic objectives for research in the European Community.

In line with our findings, studies fulfilling IHS guidelines reflect better the real efficacy of drugs. They employ more precise endpoints such as disappearance of the headache; they focus on other headache symptoms that can be as debilitating as the headache itself, and they start taking into account other aspects related with patient perspective. In our opinion, IHS guidelines should be the reference in future studies addressing headache specific treatments.

Concerning methodology, even though our study does not analyze specifically data there is an inherent risk of bias. Selection bias is possible if some studies did not have a title or were under Mesh classification. Further, in some cases it was difficult to identify the main variable or the time when it was evaluated. In those cases, we decided to leave that variable empty, which happened in 2.6% of the studies for the primary endpoint and 18.2% of the studies concerning the temporal evaluation. However, we believe the impact of this to be minimal on the final results.

## Conclusion

Headache relief has been the most employed primary endpoint; nevertheless, as IHS guidelines recommend, better efficacy endpoints like headache disappearance start to be preferred. We found a promising increase in the number of secondary endpoints, considering also disability, quality of life and patients’ preference, so future studies should subscribe them in order to warrant quality and homogeneity.

Triptans completely changed the panorama in symptomatic drug studies and their arrival improved the way RCTs are conducted in headache, nevertheless studies should subscribe guidelines in order to allow comparability as well.

To date, most of the studies took place in migraine patients but analyzing a small proportion of all the available drugs. In the future, orphan drugs and drugs with insufficient information, such as opioids, should be also evaluated and other prevalent and rare conditions should be studied in a randomized controlled setting.

## Appendix I

Command employed in the search.

(((((((((((primary headache*) OR primary headache disorder[MeSH Terms]) OR migraine) OR tension-type) OR trigeminal) OR hemicrania) OR cluster) OR sunct) OR suna) OR ""Short Lasting Unilateral Neuralgiform Headache Attacks"")) AND ((((((((telcagepant) OR ubrogepant) OR Olcegepant) OR gepant*)) OR (((((""Anti-Inflammatory Agents, Non-Steroidal""[Mesh]) OR (((((((((((((((((((naproxen*) OR aspirin*) OR salicylic*) OR salicylic) OR ((paracetamol*) OR paracetamol)) OR acetaminophen*) OR ((indomethacin*) OR Indomethacin)) OR ((dexketoprofen) OR dexketoprophen)) OR celecoxib*) OR rofecoxib*) OR diclophenac) OR ketoprofen*) OR ketorolac*) OR ibuprofen*) OR Excedrin) OR Perdolan) OR Dafalgan) OR Lonarid) OR Panadol))) OR (((((((((((domperidone) OR metoclopramide*) OR ((metamizol*) OR metamizol)) OR ((codein*) OR codeine)) OR tramadol*) OR morphin*) OR oxycodon*) OR ergot*) OR ergot) OR triptan*) OR triptan)) OR ((((lasmiditan) OR caffein*) OR magnesium*) OR ((oxygen*) OR oxygen))))) OR (((((((((((Pulsante) OR gammacore) OR Transcranial direct-current stimul*) OR springTMS) OR cefaly) OR transcranial magnetic stimul*) OR vagal nerve stimul*) OR Sphenopalatine Ganglion Stimul*) OR caloric vestibular stimul*) OR Occipital nerve stimul*) OR Transcutaneous Supraorbital Neuro Stimul*)

## Appendix II

**Table 2:** Name of the journal according to each group

		Kind of Journal			Total
		Headache J	Neuro J	General J	
	Acta Neurol Scand	0	3	0	3
	Adv Ther	0	0	1	1
	Am J Emerg Med	0	0	3	3
	Am J Med	0	0	1	1
	Ann Emerg Med	0	0	3	3
	Ann Neurol	0	1	0	1
	Arch Intern Med	0	0	2	2
	Arch Neurol	0	7	0	7
	Arq Neuropsiquiatr	0	1	0	1
	BMC Neurol	0	1	0	1
	Br J Clin Pharmacol	0	0	1	1
	Br J Prev Soc Med	0	0	1	1
	Br Med J	0	0	1	1
	Br Med J (Clin Res Ed)	0	0	1	1
	Brain Res Bull	0	1	0	1
	Cephalalgia	112	0	0	112
	Clin Drug Investig	0	0	1	1
	Clin Neuropharmacol	0	1	0	1
	Clin Pharmacol Ther	0	0	2	2
	Clin Ther	0	0	16	16
	CNS Drugs	0	5	0	5
	Curr Med Res Opin	0	0	12	12
	Drug Dev Ind Pharm	0	0	1	1
	Drug Ther Bull	0	0	1	1
	Drugs	0	0	1	1
	Eur J Neurol	0	8	0	8
	Eur J Pain	0	1	0	1
	Eur Neurol	0	19	0	19
	Expert Rev. Clin Pharmacol	0	0	1	1
	Expert Rev. Neurother	0	1	0	1
	Gynecol Endocrinol	0	0	1	1
	Headache	156	0	0	156
	Heart Dis	0	0	1	1
	Int J Clin Pract	0	0	3	3
	Int J Neurosci	0	1	0	1
	Intern Med	0	0	1	1
	J Chin Med Assoc	0	0	1	1
	J Clin Pharmacol	0	0	1	1
	J Clin Pharmacol J New Drugs	0	0	1	1
	J Coll Gen Pract	0	0	1	1
	J Emerg Med	0	0	1	1
	J Headache Pain	15	0	0	15
	J Int Med Res	0	0	6	6
	J Intern Med	0	0	2	2
	J Neurol	0	1	0	1
	J Neurol Neurosurg Psychiatry	0	1	0	1
	J Pain	1	0	0	1
	J Pharm Pharmacol	0	0	1	1
	JAMA	0	0	4	4
	Lancet	0	0	6	6
	Lancet Neurol	0	3	0	3
	Mayo Clin Proc	0	0	3	3
	MedGenMed	0	0	3	3
	N Engl J Med	0	0	2	2
	Neurol Sci	0	9	0	9
	Neurology	0	36	0	36
	Obstet Gynecol	0	0	4	4
	Pain Med	1	0	0	1
	Pain Physician	1	0	0	1
	Pain Pract	1	0	0	1
	Pediatr Neurol	0	1	0	1
	Pediatrics	0	0	3	3
	Pharmacoeconomics	0	0	5	5
	Phytother Res	0	0	1	1
	Postgrad Med	0	0	1	1
	Postgrad Med J	0	0	1	1
	QJM	0	0	1	1
	Sci Transl Med	0	0	1	1
	Singapore Med J	0	0	1	1
	Value Health	0	0	2	2
Total		287	101	107	495
