# Correction to: A PRISMA-compliant systematic review of the endpoints employed to evaluate symptomatic treatments for primary headaches

**DOI:** 10.1186/s10194-019-0967-2

**Published:** 2019-02-13

**Authors:** D. García-Azorin, N. Yamani, L. M. Messina, I. Peeters, M. A. N. Ferilli, D. Ovchinnikov, M. L. Speranza, V. Marini, A. Negro, S. Benemei, M. Barloese, on behalf of the European Headache Federation School of Advanced Studies (EHF-SAS)

**Affiliations:** 1 Headache Unit Neurology Department, Hospital Clínico Universitario Valladolid, Avda. Ramón y Cajal 3, 47005 Valladolid, Spain; 2 Danish Headache Centre and Department of Neurology, University of Copenhagen, Rigshospitalet Glostrup, Copenhagen, Denmark; 3 Headache Department, Iranian Center of Neurological Research Neuroscience Institute, Tehran University of Medical Sciences, Tehran, Iran; 4 Child Neuropsychiatry School, University of Palermo, Palermo, Italy; 5 U.O. Neuropsychiatry - ARNAS Civico, PO Di Cristina, Palermo, Italy; 6 Neurology Department, University Hospital of Brussels, Brussels, Belgium; 7 Headache Center, Bambino Gesù Children Hospital IRCCS, Rome, Italy; 8 Pavlov First Saint Petersburg State Medical University, Saint Petesburg, Russia; 9 Almazov National Medical Research Centre, Saint Petesburg, Russia; 10 Internal Medicine Department, Sant’Andrea Hospital, Rome, Italy; 11 Regional Referral Headache Centre, Department of Clinical and Molecular Medicine, Sant’Andrea Hospital, Sapienza University, Rome, Italy; 12 Headache Centre, Careggi University Hospital, University of Florence, Florence, Italy; 13 Danish Headache Center, Department of Neurology, Rigshospitalet-Glostrup, University of Copenhagen, Copenhagen, Denmark; 14 Department of Clinical Physiology and Nuclear Medicine, Center for Functional and Diagnostic Imaging, Hvidovre Hospital, Copenhagen, Denmark

**Correction to: J Headache Pain (2018) 19:90**

**https://doi.org/10.1186/s10194-018-0920-9**

Following publication of the original article [1], we have been notified that the name of author five was spelled incorrectly as **M. Ferrili**, when the correct spelling is **M.A.N. Ferilli**.
